# Effects and Mechanisms of Tea for the Prevention and Management of Diabetes Mellitus and Diabetic Complications: An Updated Review

**DOI:** 10.3390/antiox8060170

**Published:** 2019-06-10

**Authors:** Jin-Ming Meng, Shi-Yu Cao, Xin-Lin Wei, Ren-You Gan, Yuan-Feng Wang, Shu-Xian Cai, Xiao-Yu Xu, Pang-Zhen Zhang, Hua-Bin Li

**Affiliations:** 1 Guangdong Provincial Key Laboratory of Food, Nutrition and Health, Department of Nutrition, School of Public Health, Sun Yat-sen University, Guangzhou 510080, China; mengjm@mail2.sysu.edu.cn (J.-M.M.); caoshy3@mail2.sysu.edu.cn (S.-Y.C.); xuxy53@mail2.sysu.edu.cn (X.-Y.X.); 2 Department of Food Science & Technology, School of Agriculture and Biology, Shanghai Jiao Tong University, Shanghai 200240, China; weixinlin@sjtu.edu.cn; 3 College of Life Sciences, Shanghai Normal University, 100 Guilin Road, Shanghai 200234, China; yfwang@shnu.edu.cn; 4 Key Laboratory of Ministry of Education for Tea Science, Hunan Agricultural University, Changsha 410128, China; caishuxian@hunau.edu.cn; 5 School of Agriculture and Food, The University of Melbourne, Parkville, Victoria 3010, Australia; pangzhen.zhang@unimelb.edu.au

**Keywords:** tea, polyphenol, epigallocatechin-3-gallate, diabetes mellitus, complication, mechanisms

## Abstract

Diabetes mellitus has become a serious and growing public health concern. It has high morbidity and mortality because of its complications, such as diabetic nephropathy, diabetic cardiovascular complication, diabetic neuropathy, diabetic retinopathy, and diabetic hepatopathy. Epidemiological studies revealed that the consumption of tea was inversely associated with the risk of diabetes mellitus and its complications. Experimental studies demonstrated that tea had protective effects against diabetes mellitus and its complications via several possible mechanisms, including enhancing insulin action, ameliorating insulin resistance, activating insulin signaling pathway, protecting islet β-cells, scavenging free radicals, and decreasing inflammation. Moreover, clinical trials also confirmed that tea intervention is effective in patients with diabetes mellitus and its complications. Therefore, in order to highlight the importance of tea in the prevention and management of diabetes mellitus and its complications, this article summarizes and discusses the effects of tea against diabetes mellitus and its complications based on the findings from epidemiological, experimental, and clinical studies, with the special attention paid to the mechanisms of action.

## 1. Introduction

Diabetes mellitus, one of the most common metabolic disorders in the world, is featured by hyperglycemia caused by either decreased insulin secretion or insulin resistance [1]. The incidence of diabetes mellitus in adults has been increasing in the last decades [2,3]. Diabetes mellitus has been the fifth leading cause of death in the world, and the International Diabetes Mellitus Federation predicted that 592 million people worldwide will suffer from diabetes mellitus by the year 2035 [4]. Numerous studies have demonstrated that diabetes mellitus, especially type 2 diabetes mellitus (T2DM), could induce diverse complications, such as diabetic nephropathy, diabetic cardiovascular complications, neuropathy, eye, and liver complications, which have been the major causes of its morbidity and mortality [4,5]. Therefore, it is necessary and urgent to find effective strategies for the prevention and management of diabetes mellitus and its complications [6].

**Figure 1:**
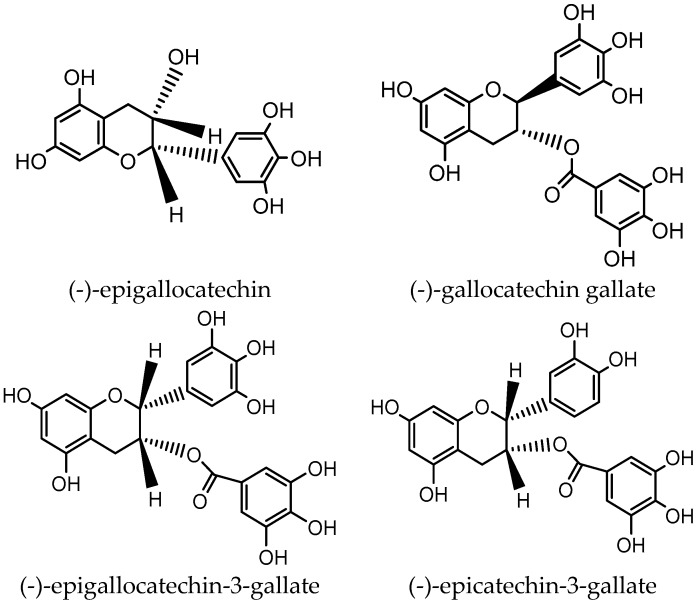
Chemical structures of main catechins in tea.

As a popular drink worldwide, tea has many bioactivities and healthy benefits, such as antioxidant, anticancer, hepatoprotective, cardioprotective, anti-obesity, improving intestinal flora, and antidiabetic effects [7,8,9,10,11,12,13,14]. Due to the different characteristics and fermentation degrees caused by various manufacturing processes, tea can be classified into six main categories, including unfermented green tea, slightly fermented white tea, partly fermented yellow tea, semi-fermented oolong tea, fully fermented black tea, and post-fermented dark tea [15,16]. Moreover, tea contains many bioactive components, especially polyphenols, such as catechins (Figure 1), flavonols, theaflavins, and thearubigins, which have the potential to decrease the risk of diabetes mellitus and its complications [17].

The effects of tea against diabetes mellitus and its complications have been widely studied. Epidemiological research found that the consumption of tea was negatively associated with the risk of diabetes mellitus and its complications [18,19]. Further, recent in vitro, in vivo, and clinical trials further supported the effects of tea on the prevention and treatment of diabetes mellitus and its complications [20,21]. In addition, tea is a potential hypoglycemic substance with low cost, good patient compliance, and fewer side effects compared with many synthetic hypoglycemic drugs [22]. In order to provide an updated understanding of tea targeting diabetes mellitus and its complications, we searched related literature of epidemiological, experimental, and clinical studies based on PubMed and Web of Science databases and reviewed and discussed the protective effects of tea against diabetes mellitus and its complications, highlighting the related molecular mechanisms.

## 2. Epidemiological Investigations

Concerning the wide consumption of tea, a number of epidemiological studies have evaluated the effects of tea on diabetes mellitus and its complications.

Several cohort studies have estimated the effects of tea on diabetes mellitus. A cohort study pointed out that the consumption of tea showed protective effects against T2DM (RR = 0.55, 95% CI (0.55, 1.08)) for Vietnamese adults [23]. Another prospective cohort study revealed a negative relationship between the risk of diabetes mellitus and the consumption of tea (HR = 0.77, 95% CI (0.59, 1.00)) for subjects aged less than 60 years old in the United States [24]. It was also reported that tea intake was negatively associated with diabetes mellitus (HR = 0.66, 95% CI (0.61, 1.22) in British subjects [25]. In addition, a prospective cohort study demonstrated an inverse association between green tea intake and the risk of diabetes mellitus (*p*-trend = 0.02) only for women in the Japanese population [26]. Furthermore, the Singapore Chinese health prospective study found that more than one cup of black tea per day could reduce the risk of diabetes mellitus by 14% (RR = 0.86, 95% CI (0.74, 1.00)) [27]. A retrospective cohort study found that green tea was inversely associated with T2DM (OR = 0.67, 95% Cl (0.47, 0.94)), while black tea and oolong tea showed no significant effects for Japanese adults [28]. For black tea, the reasons of inconsistent results may be related to the differences of dose and frequency of consumption as well as research subjects [27,28].

Some case-control studies also have evaluated the effects of tea on diabetes mellitus. For instances, a case-control study found that tea could reduce the risk of T2DM (OR = 0.66, 95% Cl (0.49, 0.89)) for Vietnamese adults [29]. Another case-control study revealed that long-term drinking of green tea had preventive effects on diabetic retinopathy (OR = 0.49, 95% CI (0.26–0.90)), and people who regularly drink green tea had a 50% lower risk of developing diabetic retinopathy than those who don’t drink green tea in China [30]. A population-based case-control study in Shantou, China found that long-term consumption of oolong tea could reduce the risk of hypercholesterolemia and triglycerides (OR = 0.10, 95% CI (0.06–0.16)) in southern China [31]. Additionally, one descriptive study revealed that long-term tea intake had negative relation with T2DM in Cyprus [32]. 

Moreover, several meta-analyses also supported the protective effects of tea consumption against diabetes mellitus. These meta-analyses included cohort studies conducted in different countries, such as America, China, Japan, and South Korea, and involved subjects of different races, genders, and ages. It was found that tea could increase the fasting blood insulin level (1.30 U/L, 95% CI (0.36–2.24)) [6], and tea consumption ≥ four cups per day could reduce the risk of T2DM (RR = 0.8, 95% CI (0.7, 0.93)) [18]. Further, another meta-analysis revealed that three to four cups of tea per day had an approximately 20% lower risk of diabetes mellitus than no tea per day (RR = 0.82, 95% CI (0.73, 0.94)) [33]. In addition, a meta-analysis including sixteen cohorts revealed a significant linear and inverse association between tea consumption and the risk of T2DM (*p* = 0.02) [34], and it was also found that more than three cups of tea per day is beneficial for the prevention of T2DM (RR = 0.84, 95% CI (0.73, 0.97) [35].

However, there are also some inconsistent results in epidemiological investigations. For instance, a prospective cohort study reported that the consumption of tea was not associated with T2DM [19]. In addition, compared with those not consuming oolong tea, multivariable adjusted hazard ratios (HR) for developing diabetes mellitus were 1.64 (95% CI (1.11–2.40)) for those drinking two or more cups of oolong per day [36]. The reasons of negative findings may be due to different sensitivities of different ethnic groups to tea, differences of tea composition in different regions, and bad control of drinking time and dose. Further, green tea was found no effects on T2DM in two meta-analysis [37,38], which may be related to the quality of the included studies, the level of evidence, and the size of the sample.

In summary, many epidemiological evidences have supported the efficiency of tea consumption against diabetes mellitus and its complications (Table 1), although inconsistent findings also exist. In addition, studies are still lacking concerning about the association between tea and diabetic complications, which should be further investigated in the future.

## 3. Experimental Studies

The effects of tea on diabetes mellitus and its complications have been widely studied by in vitro and in vivo experimental studies, and the relative mechanisms of action have also been widely explored.

### 3.1. Diabetes Mellitus

#### 3.1.1. Type 1 Diabetes Mellitus (T1DM)

T1DM is characterized by progressively destroyed pancreatic β-cells and reducing or no insulin secretion [17], accounting for 5–10% diabetes mellitus [42]. Increasing studies have found the effects of tea against T1DM [43,44,45].

The main effective catechin of green tea is epigallocatechin gallate (EGCG) [46]. EGCG could protect the functions of pancreatic β-cells by inhibiting inflammatory factors and reducing reactive oxygen species (ROS) in vitro [47]. Further, EGCG could down-regulate the production of inducible nitric oxide synthase (iNOS) to protect pancreatic islet β-cells [48]. In addition, green tea was also found to reduce blood sugar level by promoting pancreatic β-cells to produce more insulin in diabetic mice [49]. Furthermore, dark tea containing gallic acid, a water-soluble ingredient, could promote skeletal muscle glucose transport in the absence of insulin by stimulating protein kinase B (Akt) phosphorylation [50].

#### 3.1.2. Type 2 Diabetes Mellitus (T2DM)

T2DM is defined as insulin resistance in the target tissue and a relative lack of insulin secreted by islet β-cells [51], accounting for 90–95% diabetes mellitus [42]. Increasing studies showed that tea was effective in preventing and managing T2DM [6]. Next, the molecular mechanisms of tea against T2DM are discussed according to the types of tea.

A type II arabinogalactan, 7WA, isolated from green tea, could increase glucose-stimulated insulin secretion through cyclic adenosine monophosphate-Akt (cAMP-Akt) pathway [44]. Additionally, green tea polyphenols, primarily EGCG, could activate the 5‘-adenylic acid-activated protein kinase (AMPK) pathway to improve the closure of insulin stress signal pathway caused by phosphorylation of insulin receptor substrate-1 (IRS-1), finally ameliorating the insulin resistant status of human hepG2 hepatoma cells [52]. In addition, it was reported that supplement of green tea polyphenols could improve insulin sensitivity by upregulating the insulin signaling protein levels in insulin-resistant rats [53]. Moreover, green tea catechins, especially EGCG, could improve insulin resistance by scavenging ROS, which was able to block the transduction of insulin signal and prevent IRS-1 from binding to insulin receptor by decreasing tumor necrosis factor (TNF)-α-induced c-jun NH2-terminal kinase (JNK) phosphorylation [48,54]. Furthermore, EGCG played an insulin-like role in down-regulating the gene and protein expression of hepatocyte nuclear factor (HNF4), a key transcription factor controlling gluconeogenesis enzymes, such as phosphoenolpyruvate carboxykinase and glucose-6-phosphatase [55]. Moreover, green tea catechins could promote adipocyte differentiation and increase insulin sensitivity by directly activating peroxisome proliferator-activated receptor γ (PPARγ) [56]. EGCG-enriched green tea extract could also prevent T2DM by stimulating the production of soluble receptors for advanced glycation of end products (sRAGE) through a disintegrin and metallopoteases10 (ADAM10)-induced ectodomain shedding of extracellular RAGE [57].

Black tea regularly had antioxidant and anti-inflammatory effects [58] which could exert effects against T2DM. Black tea, abundant in theaflavins (accounting for 68.4% tea polyphenols), played a hypoglycemic role by inhibiting the action of ROS, such as singlet oxygen, superoxide, and hydroxyl radicals [20]. Further, black tea could reduce the risk of T2DM by inhibiting obesity through the phosphorylation of key metabolic regulator AMPK and promoting the browning of white adipose tissue [59].

Studies found that white tea had higher levels of tea polyphenols and better antioxidant activity than black tea [60]. White tea could exhibit antidiabetic activity by reducing insulin resistance, hyperlipidemia, and oxidative stress [61]. Additionally, white tea lowered blood sugar level by increasing insulin sensitivity and the synthesis of liver glycogen in T2DM rats [62]. Moreover, it was revealed that the combination of white tea and moringa oleifera had a good hypoglycemic effect [63].

It was found that the water extract of pu-erh tea contained less polyphenols but more caffeine, which could improve insulin sensitivity [5,64]. An in vitro study found that qingzhuan tea (a type of dark tea) had an inhibitory effect on α-glucosidase, which was attributed to EGCG and gallocatechin gallate (GCG) [65]. Pu-erh tea polysaccharides was also reported to regulate postprandial blood sugar by inhibiting α-glucosidase but have no effect on α-amylase activity, with older pu-erh tea exhibiting a higher inhibitory effect [66]. Moreover, it was found that ripened pu-erh tea had a better effect than raw pu-erh tea on the control of postprandial blood glucose in T2DM mice [67]. Additionally, pu-erh tea polysaccharides promoted adipocyte differentiation and glucose uptake by mimicking the properties of PPARγ and glucose transporter type 4 (GLUT4), ameliorating insulin resistance and lowering blood sugar [68]. Furthermore, Fu brick tea attenuated insulin resistance by down-regulating signal regulatory protein-α (SIRP-α) expression and activating insulin signaling in a Akt/GLUT4/FoxO1 and the target of rapamycin (mTOR)/S6K1 pathways in the skeletal muscle of male Sprague−Dawley rats [69].

Yellow tea could ameliorate glucose intolerance and insulin resistance without dose dependence [70]. Further, the roasted yellow tea could improve insulin sensitivity and reduce fasting blood sugar due to the strong affinity of GCG to target protein-glycosidase, and the strong inhibition effect of GCG on α-glucosidase activity [71].

In general, different types of tea exhibit antidiabetic effects in vitro and in vivo. Tea catechins, theaflavins, polysaccharides, and caffeine should be mainly responsible for the antidiabetic effects of tea. Notably, these bioactive compounds in tea can regulate signal pathways and key molecules involved in the regulation of insulin, blood sugar, and energy metabolism.

### 3.2. Diabetic Complications

#### 3.2.1. Diabetic Nephropathy

Diabetic nephropathy is one of the major microvascular complications of diabetes mellitus [72]. Its obvious pathological changes were persistent proteinuria, changes in creatinine clearance, mesangial matrix dilatation, thickening of glomerular basement membrane, and glomerular sclerosis [73]. Numerous studies demonstrated that tea could ameliorate the pathological process of diabetic nephropathy through the antioxidant and anti-inflammatory properties [74,75]. The effects and related molecular mechanisms of tea against diabetic kidney injury are discussed below.

Green tea extract could provide a beneficial effect against long-term diabetic nephropathy via suppressing hyperglycaemia, preventing glycogen accumulation in the proximal tubules, and improving serum and urine parameters (e.g., glucose, glycosylated proteins and creatinine, and blood urea nitrogen) [70]. Green tea could defend renal tubular by reducing the urinary activity of renal tubular epithelial-cell enzymes [76]. Furthermore, green tea catechins could protect kidney function by reducing the permeability of glomerular filtration membrane through inhibiting thrombosis with lowered microsomal phospholipase A2 and regulating arachidonic acid cascade system [42]. An in vivo study also found that green tea could improve glomerular filtration and reduce the rate of creatinine increase and renal hypertrophy [77]. Further, in diabetic spontaneously hypertensive rats (SHRs), green tea prevented podocyte apoptosis and albuminuria by rising p-low-density lipoprotein receptor-related protein 6 (p-LRP6) expression and blocking glycogen synthase kinase 3 interaction with p53 (GSK3-p53) [78]. Another study also found green tea polyphenols could attenuate the urinary protein excretion and characteristic morphological changes of diabetic nephropathy by decreasing blood glucose levels [79]. Additionally, (+)–catechins might ameliorate renal dysfunction in diabetic mice by inhibiting advanced glycation end product (AGE) formation and cutting off inflammatory pathways via trapping metabolite methylglyoxal [80]. Also, green tea catechins could reduce the oxidative damage and inflammatory reaction in the kidney by regulating the activity of 5′-lipoxygenase and inhibiting the generation of superoxide radicals, oxidative proteins, lipids, and leukotriene B-4 in the kidneys of diabetic rats [81]. Moreover, green tea flavonoids could reduce ROS via three pathways, including the activation of PPARγ in the eukaryotic elongation factor-2 kinase (EEF2K) pathway by enhancing 5′AMPK, the induction of nuclear factor-erythrocyte-associated factor 2 (Nrf2), by activating the Kelch-like ECH-associated protein 1-antioxidant-responsive element (Keap1-ARE) signaling, and the regulation of Mn superoxide dismutase production via Forkhead box O3 (FOXO3) -Akt pathway by increasing sirtuin-1 [82]. In addition, green tea catechins, especially EGCG and epicatechin gallate (ECG), could improve the thickening of the basement membrane by relieving the damage of matrix metalloproteinase (MMP), which could degrade extracellular matrix and fibrosis, finally alleviating diabetic nephropathy [83,84,85].

Besides green tea, other types of tea also show protective effects against diabetic nephropathy. Pu-erh tea could ameliorate diabetic nephropathy by decreasing RAGE expression and glomerular IgG deposit through inhibiting AGE accumulation [73]. Cuiyu tea (a dark tea) polypeptides were reported to stimulate the PKCζ/JNK/nuclear factor-κB (NF-κB)/THF-α/iNOS, advanced glycation end products (AGEs)/RAGE/TGF-1 pathway, up-regulate the expression of podocin in glomeruli, and decrease the release of proinflammatory cytokines, thereby ameliorating diabetic nephropathy [86]. Further, oolong tea polysaccharides could reduce renal tissue inflammation and improve the glomerular vascular permeability of glutathione peroxidase (GSH-PX) by enhancing the activity of superoxide dismutase and GSH-PX [87]. In general, tea exhibits good effects against diabetic nephropathy in vitro and in vivo.

#### 3.2.2. Diabetic Cardiovascular Diseases

Cardiovascular complications, containing cardiomyopathy, atherosclerosis, coronary ischemia, and vascular disease, are associated with the high morbidity and mortality of diabetes mellitus [77,88], among which cardiomyopathy is responsible for 80% [17]. It was reported that tea could improve cardiovascular complications by decreasing hyperglycemia, adjusting lipid metabolism, activating signaling pathways, down-regulating the inflammatory factors, and so on.

Tea polyphenols could improve myocardial glycolipid energy metabolism and interfere with adiponectin mRNA and protein expression through AMPK activation, which was involved in insulin signaling [89]. Also, tea polyphenols could stimulate the activity of AMPK via the Ca^2+^/Ca-MKK/AMPK-mediated signaling pathway [90]. Additionally, tea polyphenols could regulate autophagy via mTOR and Akt signaling pathways, which was beneficial for the prevention and treatment of diabetic cardiomyopathy [91,92,93].

An in vivo study found that green tea could inhibit cardiac dyslipidemia, lipid peroxidation, and protein glycosylation through improving the activities of Ca^2+^-ATP and Na^+^/K^+^-ATP enzymes to regulate the content of Ca^2+^ and Na^+^ [94]. Furthermore, EGCG could improve metabolic and cardiovascular pathophysiology by stimulating the production of nitric oxide (NO) from endothelium via phosphatidylinositol 3-hydroxykinase (PI3k)-dependent pathways [95]. Additionally, EGCG could inhibit inflammatory factors such as NF-κB and activator-1, which played an important role in vascular inflammation caused by insulin resistance [96,97]. In addition, it was found that green tea extract could reduce the progression of atherosclerosis by reversing endothelial dysfunction [98]. Also, MMP-9 played an important role in atherosclerosis, and green tea catechins could inhibit the mRNA expression and the activity of MMPs [83,99]. Further, green tea catechins increased plasma total antioxidant activity and prevented diabetic cardiovascular autonomic dysfunction by blocking changes in arterial pressure variability [77]. Moreover, the green tea extract inhibited the accumulation of aortic collagen, reduced the solubility of collagen, and decreased AGEs and collagen cross-linking, finally preventing diabetic cardiomyopathy in streptozotocin diabetic rats [98,100]. It was also reported that green tea flavonoids could alleviate the contraction of aortic strips, and green tea extract could protect against free radical and glucose-mediated protein damage [101].

Both black tea theaflavins and green tea catechins significantly attenuated high glucose-induced block of insulin signaling, reduced lipid accumulation, inhibited fatty acid synthesis, and stimulated fatty acid oxidation by activating the LKB1-AMPK pathway [54,102,103]. Furthermore, insulin deficiency and insulin resistance could induce exaggerated vasoconstriction [104], and black tea polyphenols could improve vasoconstriction through PI3K-Akt pathway and endothelial nitric oxide synthase (eNOS) phosphorylation [105,106].

White tea catechins could reduce the absorption of cholesterol in the intestines and increase the excretion of cholesterol and total fat in the feces [107]. Further, white tea could control the Krebs cycle by decreasing pyruvate through regulating lactic acid and the lactate dehydrogenase levels in the heart at the prediabetic state, thereby preventing diabetic cardiovascular disease [108,109,110]. In addition, white tea could regulate cardiac metabolic disorders by up-regulating the expression of cardiac GLUT1 and GLUT3 mRNA [87]. Quercetin, an important flavonoid found in write tea, could prevent diabetic vascular complications in both insulin deficiency and resistance by inhibiting inflammatory pathways, especially the NF-κB signaling [104].

Yellow tea “Junshan Yinzhen” could improve insulin resistance and the disorders of glucose and lipid metabolism in diabetic rats, which may be related to the high content of polyphenols, polysaccharides, and alkaloids in yellow tea [111]. Besides, yellow tea could control glucose effectively by maintaining normal expression of thioredoxin interacting protein, which played an important role in the synthesis and release of glucose in the liver [112].

Oolong tea contained less catechins than green tea, but more catechins than black tea [113]. Pu-erh tea extract could inhibit inflammation of visceral adipose tissue by down-regulating the inflammatory factors and inducing the expression of G-protein coupled receptor (Gpr120) [114].

Therefore, tea and its bioactive components, can be good protectors for cardiovascular complications of diabetes.

#### 3.2.3. Diabetic Neuropathy

Neurodegeneration is characterized by increased free radical production and oxidative stress [115]. It has been proved that tea had neuroprotective effects due to its anti-inflammatory and antioxidant properties [116]. An in vivo study found that green tea could prevent autonomic nervous dysfunction by blocking the change of arterial pressure variability [77]. Furthermore, green tea extract could restore the analgesic effect of morphine on diabetic neuropathic pain by inhibiting the production of NO [117]. Further, EGCG could reduce the formation of neural tube defects in embryo caused by maternal diabetes mellitus in mice through blocking the expression and activity of DNA methyltransferase, and inhibiting DNA hypermethylation, and restoring the expression of neural tube closure essential gene [118]. However, the protective effects of tea other than green tea on diabetic neuropathy have been much less investigated, and it could be interesting to investigate whether other types of tea can be also effective to protect against diabetic neuropathy.

#### 3.2.4. Diabetic Retinopathy

Diabetic retinopathy is a common microvascular complication of diabetes mellitus [119]. Hyperglycemia, oxidative stress, and advanced glycation end products are all risk factors of diabetic retinopathy [120]. Tea has shown protective effects on diabetic retinopathy. In diabetic rats, EGCG could protect the retina by decreasing the level of anion and preventing the formation of acellular capillaries and pericyte ghosts [121]. Also, green tea could protect the nerves of diabetic retinas and regulate the subretinal environment by reducing the production of ROS by increasing glutamate transporter expression, reestablishing intercellular connections, and restoring glutamine/glutamate circulation [122]. In addition, green tea at very low dose could improve antioxidant defense, reduce inflammatory markers, and prevent retinal basement membrane thickening [123]. Further, black tea could delay the development of diabetic cataracts by lowering blood sugar, thereby inhibiting pathological biochemical indicators [124]. Oolong tea extract could increase plasma retinol levels in diabetic rats [125]. Overall, tea acts as a potent neuroprotector in diabetic retinas.

#### 3.2.5. Diabetic Hepatopathy

Diabetic hepatopathy is potentially less common [126] but tends to be more prevalent among children [127]. Several studies suggested that tea and tea polyphenols could play important roles in diabetic liver injury through their antioxidant and anti-inflammatory activities [128,129].

In apparently healthy individuals, green tea extract could reduce oxidative stress and reduce the risk of diabetic liver diseases by lowering the malondialdehyde level and increasing the glutathione level [126]. In addition, green tea could ameliorate liver inflammation and damage caused by diabetes mellitus by lowering angiotensin II receptors [130]. Further, black tea extract protected the liver by increasing cellular antioxidant capacity and reducing membrane lipid peroxidation, inhibiting oxidative stress in diabetic and obese rats caused by alloxan and high cholesterol diets [17]. A dietary supplement of yellow tea and write tea regulated glucose and lipid metabolism by reducing the expression of fatty acid synthase, a membrane surface molecule leading to cell apoptosis, and sterol response element-binding protein 1 in db/db mice [15].

**Figure 2:**
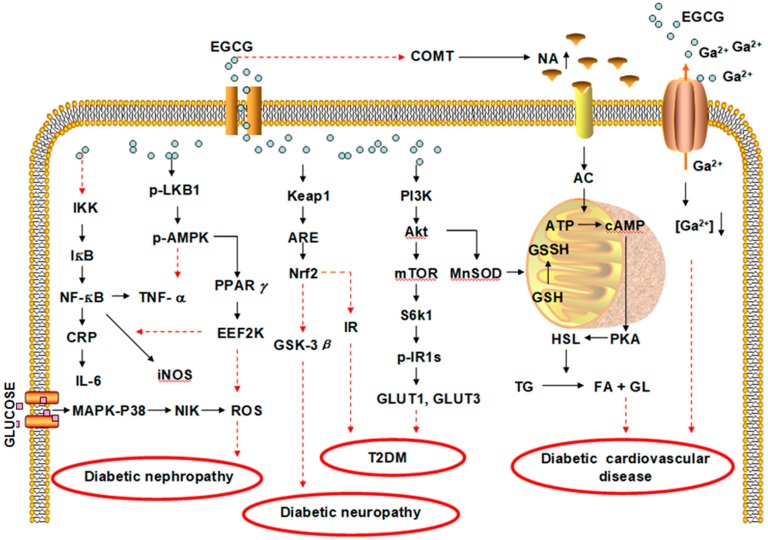
The molecular mechanisms of EGCG against diabetes mellitus and its complications. EGCG has shown effects against T2DM by improving IR, against diabetic cardiovascular disease by decreasing TG and [Ga^2+^], against diabetic nephropathy by decreasing ROS and against diabetic neuropathy by increasing Nrf2. The arrow means the direction of actions, and the black full lines indicate upregulation and red dotted lines refer to downregulation or inhibition. CRP, C-reactive protein; MAPK p38-NIK, NF-κB inducing kinase; LKB1, kelch-like ECH-associated protein-1; EEF2K, eukaryotic elongation factor-2 kinase; ARE, antioxidant-responsive element; GSK-3β, glycogen synthase kinase-3β; IR, insulin resistance; MnSOD, Mn superoxide dismutase; NA, noradrenalin; s6k1, ribosomal protein S6 kinase 1; AC, adenylate cyclase; HSL, hormone-sensitive lipase; TG, triglyceride; FA, fatty acid; GL, glycerinum; GSH, glutathione; GSSH, oxidized glutathione; mTOR, the target of rapamycin; EGCG, epigallocatechin gallate; IKK, IκB kinase; NF-κB, nuclear factor-κB; iNOS, inducible nitric oxide synthase; TNF-α, tumor necrosis factor-α; Nrf2, nuclear factor-erythrocyte-associated factor 2; PI3K, phosphatidylinositol 3-hydroxykinase; Akt, protein kinase B; AMPK, adenylic acid-activated protein kinase; T2DM, type 2 diabetes mellitus; GLUT, glucose transporter type; PKA, protein kinase A; ATP, adenosine triphosphate; cAMP, cyclic Adenosine monophosphate; COMT, catechol-O-methyltransferase, an enzyme responsible for the degradation of noradrenalin.

Notably, the molecular mechanisms of EGCG on diabetes mellitus and its complications are summarized in Figure 2.

#### 3.2.6. Other Complications

Tea is also beneficial for other diabetic complications, such as osteoporosis, periodontal disease, and reproductive dysfunction. For example, green tea could treat periodontal disease by decreasing the expression of pro-inflammatory cytokine TNF-α and the osteoclastogenic mediator receptor activator of the NF-κB ligand (RANKL) and up-regulating the expression of anti-inflammatory cytokine interleukin-10, osteogenesis-related factor runt-related transcription factor 2 (RUNX-2), and anti-osteoclastogenic factor osteoprotegerin (OPG) [131]. Moreover, epigallocatechin (EGC) had a positive effect on bone metabolism by promoting osteoblast activity and inhibiting osteoclast differentiation [132].

### 3.3. Adjuvant Therapy

Currently, acarbose, rosiglitazone, and metformin are the main antidiabetic drugs used in clinics [66,133]. Several studies reported that tea had synergistic effects with antidiabetic drugs on diabetes and its complications, making tea a promising adjuvant for diabetes treatment. It was shown that pu-erh tea could enhance the effect of rosiglitazone on antidiabetic nephropathy by preventing the diabetes-induced accumulation of AGEs and re-establishing a normal RAGE level in vivo [69]. Black tea and acarbose also showed a mixed-type effect on the modulation of postprandial hyperglycemia by inhibiting the α-glucosidase activity in the small intestine [134]. Low concentrations of green tea polyphenols or EGCG had a synergistic effect with acarbose on α-amylase and α-glucosidase in vitro [135].

**Figure 3:**
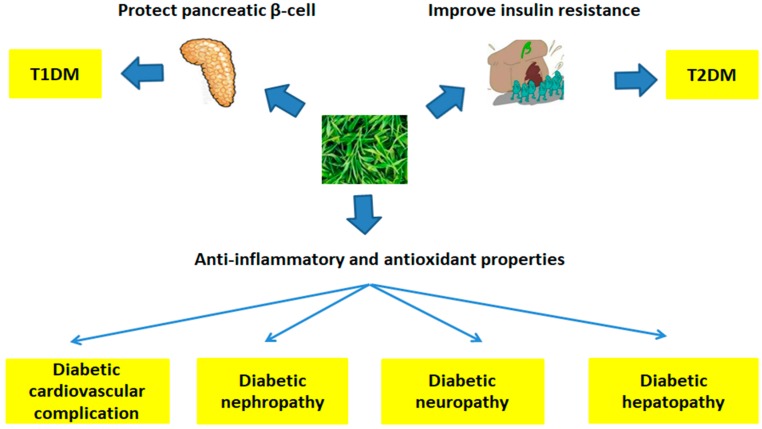
The association between tea and diabetes and its complications. Tea has effects on type 1 diabetes mellitus (T1DM) and type 2 diabetes mellitus (T2DM) by protecting pancreatic β-cells and ameliorating insulin resistance. Besides, due to the anti-inflammatory and antioxidant properties of tea, diabetic complications, including diabetic cardiovascular complication, diabetic nephropathy, diabetic neuropathy, and diabetic hepatic tissue injury, could be prevented and treated by tea and its bioactive components.

To sum up, a great number of studies indicated that tea and its bioactive components could be used for the prevention and treatment of diabetes mellitus and its complications (Table 2). The main mechanisms of action included protecting pancreatic β-cells, ameliorating insulin resistance, anti-inflammatory, and antioxidant potentials (Figure 3). Furthermore, tea showed a synergistic effect with certain antidiabetic drugs on diabetes and its complications.

## 4. Clinical Trials

Several clinical trials have assessed the role of tea in treating diabetes mellitus and diabetic complications.

A double-blind, randomized, controlled clinical trial (RCT) found that green tea could improve bone mineral levels in patients from Brazil with diabetes mellitus [153]. Another RCT observed that green tea extract significantly reduced bone resorption markers and altered bone conversion in T2DM patients [154]. Furthermore, a phase I clinical trial involving 63 patients with T2DM found that drinking 4 cups of green tea per day for two months significantly reduced body weight and systolic blood pressure [155]. Another RCT conducted in Taiwan observed that green tea extract significantly improved insulin resistance and increased glucagon-like peptide 1 [156]. It was proven that green tea could reduce risk factors of diabetes mellitus such as average arterial pressure, waist-to-hip ratio, and glutamic-pyruvic transaminase, but had little effect on fasting blood sugar and hemoglobin A1c (HbA1c), which may be related to the short time span of the study, for subjects in Mauritius [21]. Another RCT conducted in British showed that green tea extract could reduce proteinuria in diabetic patients [41]. Further, it was reported that postprandial blood sugar could be decreased in people drinking black tea [39]. A clinical trial conducted in Kuwait also revealed that drinking black tea for one year could significantly reduce HbA1c level and pro-inflammatory CD3^+^ CD4^+^ IL-17^+^ cells, and eliminate serum total cholesterol, thereby preventing diabetes mellitus and its complications [22]. Moreover, another trial demonstrated that black tea could protect against diabetes mellitus and diabetic cardiovascular disease through its anti-inflammatory and antioxidant properties [58]. Oolong tea was proven to be an effective adjunctive oral hypoglycemic substance for T2DM in a clinical trial in diabetic patients from Taiwan taking normal hypoglycemic drugs [157].

In brief, clinical trials involving different countries and different people showed that tea could prevent and manage diabetes mellitus and its complications, mainly by improving insulin resistance and decreasing postprandial blood sugar (Table 3).

## 5. Conclusions

Diabetes mellitus and its complications have become an important public health problem. Epidemiological studies found that drinking tea could reduce the risk of diabetes mellitus and diabetic complications, and among these studies, green tea, black tea, and oolong tea were in the majority, while epidemiological studies on white tea, dark tea, and yellow tea were less common. In addition, experimental studies have shown that tea could protect against diabetes mellitus and diabetic complications by improving insulin resistance, activating the insulin signaling pathway, playing an insulin-like role, improving oxidative stress, and alleviating inflammatory response. Further, tea has synergistic effects with certain antidiabetic drugs. Moreover, clinical trials have shown that tea played a positive role in the prevention and treatment of diabetes mellitus and its complications. Additionally, different types of tea have different main bioactive ingredients, which may be applicable to different diabetic complications. Therefore, tea could be used as a beverage, or be developed into functional foods or nutraceuticals, for the prevention and management of diabetes mellitus and its complications, such as diabetic nephropathy, diabetic cardiovascular disease, and diabetic retinopathy. In the future, more bioactive components in tea for the prevention and management of diabetes mellitus and its complications should be separated and identified, especially for the dark tea. The molecular mechanisms of tea and its bioactive components should be further studied. In addition, because of the differences of doses and effects of tea between experimental and clinical studies, it is still difficult to conclude whether the effective doses from animal studies might have beneficial effects on human. Therefore, more clinical trials should be carried out to verify the protective effects of tea on diabetes mellitus and its complications. In addition, special attention should be paid to the safety of tea and tea products.

## Figures and Tables

**Table 1:** The relationship of tea consumption and diabetes mellitus by epidemiological studies.

Diseases	Tea Type	Study Type	Participants	Dose	Results	Ref.
Diabetes mellitus	Tea	Prospective cohort study	Individuals (N = 7006) aged 32–88 without diabetes mellitus	N/A	The consumption of tea showed an decreased risk of diabetes mellitus for nonelderly adults who had previously lost weight.	[24]
Diabetes mellitus	Green tea	Cohort study	Elderly Japanese men and women (N = 11,717)	N/A	Women with a higher intake of green tea had a lower risk of diabetes mellitus.	[26]
Diabetes mellitus	Black Tea	placebo-controlled study	Total participants (N = 24) aged 20–60	N/A	Drinking black tea could decrease postprandial blood sugar.	[39]
T2DM	Tea	Population-based cohort study	Danish non-diabetic women with singleton pregnancies (N = 71,239)	8 cups per day	The consumption of tea showed protective effects against T2DM (RR = 0.55, 95% CI (0.55, 1.08)).	[23]
T2DM	Tea	Prospective Cohort study.	African American women (N = 46,906)	N/A	The consumption of tea wasn’t associated with T2DM.	[19]
T2DM	Tea	Prospective cohort study.	British men (N = 4055) and women (N = 1768)	N/A	Tea intake was beneficial for DM (HR = 0.66, 95% CI (0.61, 1.22)), *p* < 0.05.	[25]
T2DM	Tea	Case-Control study	Newly diagnosed diabetic cases (N = 599) Hospital-based controls (N = 599)	2 cups per day	Habitual drinking tea could reduce the risk of T2DM (OR = 0.66, 95% CI (0.49–0.89)).	[29]
T2DM	Tea	Case-Cohort Study	Total participants (N = 16,835)	≥1 cups per day	The consumption of tea has negative relation with T2DM 1 cup/day (HR = 0.84, 95% CI (0.71, 1.00)) ≥2 cups/day (HR = 0.93, 95% CI (0.81, 1.05))	[40]
T2DM	Tea	Meta-analysis	Total participants (N = 545,517); Cases with T2DM (N = 37,445)	N/A	The consumption of tea has negative relation with T2DM (*p* = 0.02).	[34]
T2DM	Tea	Meta-analysis	N/A	N/A	Drinking tea daily (≥3 cups/day) is associated with a lower risk of T2DM (RR = 0.84, 95% CI (0.73, 0.97))	[35]
T2DM	Tea	Meta-analysis	Total participants (N = 324,141); Cases with T2DM (N = 11,400)	N/A	Tea consumption a ≥4 cups per day may lower the risk of T2DM.	[18]
T2DM	Tea	Meta-analysis	Total participants (N = 457,922)	N/A	The consumption of tea was associated with reduced risk of diabetes mellitus.	[33]
T2DM	Tea	Descriptive study	Total participants (N = 940)	N/A	Long-term tea intake had effects on the prevention and treatment of diabetes mellitus.	[32]
T2DM	Green tea	Meta-analysis	N/A	N/A	The consumption of tea wasn’t associated with T2DM.	[38]
T2DM	Green tea	Meta-analysis	N/A	N/A	Tea or tea extract could maintain stable fasting insulin level in patients with T2DM.	[6]
T2DM	Green tea	Meta-analysis	Total participants (N = 510)	N/A	Green tea had no effect on insulin sensitivity and blood glucose control.	[37]
T2DM	Black tea	Cohort study.	Total participants (N = 36,908)	≥1 cups per day	Black tea had association with T2DM (RR = 0.86, 95% CI (0.74, 1.00)).	[27]
T2DM	Oolong tea	Prospective cohort study.	Japanese male workers (N = 4975)	≥1 cups per day	Long-term consumption of oolong tea may be a predictive factor for new onset diabetes mellitus. 1 cup/day (HR = 1.00, 95% CI (0.67–1.49)). ≥2cups /day (HR = 1.64, 95% CI (1.11–2.40))	[36]
Diabetic nephropathy	Green tea	Cohort study	Diabetic patients (N = 42)	N/A	Green tea extract could reduce proteinuria in diabetic patients.	[41]
Diabetic Retinopathy	Green tea	Case-Control Study	Cases with diabetic retinopathy (N = 100) and diabetic patients without retinopathy (N = 100)	N/A	Long-term drinking green tea had preventive effects on diabetic retinopathy (OR = 0.49, 95% CI (0.26–0.90)).	[30]

Abbreviations: T2DM, type 2 diabetes mellitus; N/A, not available.

**Table 2:** The effects of tea on diabetes mellitus and its complications by in vitro and in vivo studies.

Tea Types	Constituents	Diseases Types	Study Types	Models	Dose	Effects	Mechanisms	Ref.
Green tea	EGCG	Diabetic cardiovascular disease	In vivo	Alloxan-induced diabetic rabbits	50 mg/kg/day	Improved late endothelial progenitor cells(L-EPCs); Promoted reendothelialization.	Activated Akt/eNOS pathway	[136]
	EGCG	Diabetic cardiomyopathy	In vivo	Wistar rats	50 mg/kg/day	Enhanced cardiac function; Increased ADSC repair capability;	↑ Insulin-like growth factor 1 ↑ H9c2 cell cycle	[137]
	EGCG	diabetic neuropathy	In vivo	Male Wistar rats	0.1% (w/v)	Improved cerebral function.	↓ Neuronal degeneration ↓ Apoptotic cell death	[138]
	Polyphenols	Diabetic Retinopathy	In vivo	Wistar-Kyoto rats	5.7 g/kg/day	Protected the retina against glutamate toxicity.	↓ ROS	[122]
	Polyphenols	Diabetic cardiovascular disease	In vivo	Male Wistar rats	0.8, 1.6, and 3.2 g/L	Reduced fat deposit; Ameliorated hypoadiponectinemia in HF-fed rats; Relieved high glucose-induced adiponectin decrease.	↓ Extracellular signal regulated kinase 1/2 phosphorylation ↑ PPARγ ↓ Adiponectin decrease	[139]
	Polyphenols	Diabetic cardiovascular disease	In vitro	Cardiac muscle of rats	200 mg/kg	Ameliorated the effects of high-fructose diet on insulin signaling, lipid metabolism and inflammation.	↑ PI3k, Akt1 ↑ Glut1, Glut4, glycogen synthase 1 ↑ Anti-inflammatory protein ↓ GSK-3β, TNF, IL-1B and IL-6	[53]
		Diabetic cardiovascular disease	In vivo	STZ-induced rats	300 mg/kg/day	Protected rat heart.	↓ [Ca^2+^] and [Na^+^] ↑ Activities of Ca^2+^-ATPase and Na^+^/K^+^-ATPase	[94]
		Diabetic cardiovascular disease	In vivo	STZ-induced rats	300 mg/kg/day	Reduced the risk of diabetic cardiovascular disease.	↓ Cholesterol, triglyceride ↓ Free fatty acid and LDL-C ↑ HDL-C	[140]
		Diabetic cardiomyopathy	In vivo	Diabetic rats	300 mg/kg/day	Treated diabetic cardiomyopathy.	↓ AGEs ↓ Ollagen cross-linking	[100]
		diabetic retinopathy	In vivo	Rats	200 mg/kg/day	Prevented and treated diabetic retinopathy.	↓ SOD and catalase enzyme	[123]
		Diabetic hepatopathy	In vivo	Male Wistar rats	1.5% (w/v)	Prevented diabetic tissue injury.	↑ GSH-Px, SOD, catalase	[126]
		Diabetic hepatopathy	In vivo	Male Wistar rats	1.5% (w/v)	Pretected tissue.	↑ GSH-Px, SOD, catalase ↓ MDA, alkaline phosphatase	[141]
		Diabetic nephropathy and hepatopathy	In vivo	Male Sprague-Dawley rats	0.1% (w/v)	Protected renal and hepatic tissues from injury.	↑ Total antioxidant levels ↓ Malonyldialdehyde (MDA) ↓ Angiotensin II AT1 receptor	[130]
		Diabetes mellitus-induced periodontitis	In vivo	STZ-induced rats	N/A	Treated diabetes mellitus-induced periodontitis.	↓ TNF-α and RANKL ↑ RUNX-2, OPG ↑ Interleukin-10 (IL-10)	[131]
		diabetic spinal cord	In vivo	STZ-induced rats	N/A	Improved diabetic spinal cord.	↑ GFAP	[142]
Black tea		T1DM	In vivo	Female CD-1 mice	0.01% (w/v)	Promoted insulin secretion and regenerated damaged pancreas and protected pancreatic β- cells.	↓ Nitrosative stressRUNX-2, OPG↓ ROS	[20]
		Diabetes mellitus	In vivo	STZ-induced rats	0.5 mL/day	Regenerated damaged pancreas and protected pancreatic β-cells.	↓ Nitrosative stress	[143]
		T2DM	In vivo	STZ-induced rats	0.01 mL/g/day	Ameliorated diabetes mellitus associated oxidative stress.	↑ GSH	[144]
		Diabetic complication	In vivo	Diabetic animals	50 mg/mL	Attenuated oxidative stress mediated tissue damage.	↓ DNA fragmentation ↓ Activation of caspase-3 ↑ Oxidative stress related parameters	[108]
		Diabetic tissue injury	In vivo	Adult male Wistar albino rat	50 and 100 mg/kg/day	Protected the liver	↑ Cellular antioxidant capacity ↓ Membrane lipid peroxidation ↓ Oxidative stress	[17]
	EGC, GC, GCG	bone metabolism	In vitro	Cultured rat osteoblast-like osteosarcoma cell line UMR-106	N/A	Improved bone metabolism	↑ Osteoblast activity ↓ Osteoclast differentiation	[132]
White tea		T2DM	In vivo	Male Sprague-Dawley rats	0.5% (w/v)	Lowered blood sugar levels.	↑ Insulin sensitivity ↑ The synthesis of liver glycogen	[62]
		Diabetic cardiovascular diseases	In vivo	Male Wistar rats	0.01 mg/mL	Prevented cardiovascular diseases.	↑ Insulin sensitivity ↑ Cardiac acetate and alanine contents and protein oxidation	[88]
		Diabetes mellitus	In vitro	human hepatocellular carcinoma (HepG2) cell	25 mg/mL	Improved glucose and lipid metabolism.	↓ Glucose uptake and transport	[145]
		Diabetic reproductive dysfunction	In vivo	STZ-induced prediabetic rat model	10 mg/mL	Improved epididymal sperm motility and restored sperm viability.	↓ GLUT3 protein ↑ Lactate dehydrogenase ↑ Lactate content.	[146]
Dark tea	EGCG, ECG	Diabetes mellitus	In vitro	N/A	50 mg/mL	Treated diabetes mellitus.	↓ α-glucosidase	[65]
	TP,TPS	Diabetes mellitus	In vivo	Diabetic rats	50 mg/kg	Reduced postprandial blood sugar.	↓ α-glucosidase	[147]
	Polysaccharides	T2DM	In vivo	Male ICR mice	40 mg/kg	Lowered the blood glucose levels and reversed oxidative stress.	↑ SOD activity ↑ Malondialdehyde contents ↑ GSH-Px	[148]
		T2DM	In vivo	Male ICR mice	1 and 5 mg/kg	Improved insulin resistance.	↓ α-glucosidase Maintain α-amylase	[66]
		T2DM	In vitro In vivo	HepG2 cells db/db mice	100, 200, and 400 mg/kg/day	Improved insulin resistance and maintained glucose homeostasis.	↑ Glucose uptake ↓ Intestinal sucrase, maltase, and porcine pancreatic amylase activity	[5]
		T2DM	In vivo	Male Sprague−Dawley rats	400 mg/kg/day	Alleviated insulin resistance and chronic kidney disease.	↓ SIRP-α ↑ PI3K/Akt ↑ Nrf2 expression in kidney ↓ GSK-3β phosphorylation Activated Akt/GLUT4, FoxO1 and mTOR/S6k1 pathways	[69]
		diabetic nephropathy	In vivo	db/db mice and db/m mice	1 g/kg/day	Attenuated the increases in urinary albumin, serum creatinine, and mesangial matrix.	↓ AGEs ↓ Receptor for AGE expression in glomeruli ↓ Carbonyl compounds	[73]
Onloog tea	Polysaccharide	diabetic tissue and kidney	In vivo	STZ-induced diabetic diabetic mice	50, 100, and 200 mg/kg	Prevented diabetic tissue and kidney diseases.	↑ SOD and GSH-PX activity ↓ MDA	[87]
	Polysaccharide	Diabetic immune disease	In vivo	STZ-induced diabetic mice	100, 300, and 600 mg/kg in mice 50, 100, and 200 mg/kg in rats	Improved immunomodulatory function.	↑ The activity of NK cellsIntensify DTH ↑ Phagocytotic function of peritoneal macrophage	[149]
Yellow tea	EGCGGCG	Diabetes mellitus	In vitro	N/A	1% (w/v)	CGC reduced postprandial blood sugar more effectively.	↓ α-glucosidase	[71]
		Diabetic complications	In vivo	db/db mice	N/A	Lowered the serum total and low-density lipoprotein cholesterol and triglyceride levels. Increased glucose tolerance.	↓ The lipid synthesis ↓ SRET fator1, SREP 1 ↓ Acetyl-CoA carboxylase α, ↓ Fatty acid synthase	[15]
Tea	EGCG	T1DM	In vitro	RINm5F cells	20-40 *u*M	Protected pro-inflammatory cytokine and induced injuries in insulin-producing cells.	↓ iNOS and NO	[47]
		T1DM	in vivo	C57BL/KsJ mice	100 mg/kg/day	Protected pancreatic islets.	↓ iNOS	[150]
	EGCG	T2DM	In vivo	Diabetic patients	300, 600, and 900 mg/day	Decreased pathogenesis of proinflammation and improved diabetes mellitus.	↓ Free radicals ↓ S100A12-RAGE axis by stimulating sRAGE	[57]
	Catechins	T2DM	In vivo In vitro	Male obese KK-ay and C57BL/6J mice; 3T3-L1 adipocytes	20 mg/kg/day	Decreased glucose levels and increased glucose tolerance in animals.	↓ ROS ↓ JNK phosphorylation ↑ GLUT-4 translocation	[48]
	EGCG	T2DM	In vitro	Human HepG2 cells	N/A	Attenuated insulin signaling blockade.	↓ Phosphorylation of IRS-1 ↑ 5′AMPK	[52]
	EGCG	T2DM	In vivo	Sprague-Dawley rats	1-100 *u*M	Improved endothelial dysfunction and insulin resistance and protected against myocardial I/R injury.	↑ NO via PI3k pathway ↑ Plasma adiponectin	[95]
		diabetic nephropathy	In vivo	Diabetic SHR rats	5.7 g/kg/day	Reduced podocyte apoptosis, foot process effacement and albuminuria.	↓ GSK3-p53 ↑ LRP6	[78]
		diabetic nephropathy	In vivo	STZ-induced diabetic rats	5% (w/v)	Improved diabetic nephropathy.	↓ MMP-9, TIMP-1 ↑ MMP-2 ,TIMP-2	[83]
		diabetic nephropathy	In vivo	Male Sprague-Dawley rats	0.25% and 0.5% (w/w)	Reduced renal oxidative damage and inflammatory reactions.	↑ Activity of 5′-lipoxygenase ↓ Ieukotriene B-4	[81]
	Catechins	diabetic nephropathy	In vivo	Sprague-Dawley rats	0.25% and 0.5% (w/w)	Improved kidney function.	↓ Thromboxane A(2) synthesis ↑ Prostacyclin synthesis	[151,152]

Abbreviations: iNOS, inducible nitric oxide synthase; RANKL, receptor activator of nuclear factor kappa-B ligand; OPG, osteoprotegerin; RUNX-2, runt-related transcription factor 2; GFAP, glial fibriliary acidic protein; SRET, sterol regulatory element-binding transcription factor 1; SREP, synthase and sterol response element-binding protein; TIMP, tissue inhibitor of metalloproteinases; STZ, streptozotocin; SHR, spontaneous hypertension rat, ICR, Institute of Cancer Research; Akt, protein kinase B; eNOS, endothelial nitric oxide synthase; PPARγ, peroxisome proliferator-activated receptorγ; PI3K, phosphatidylinositol 3-hydroxykinase; GLUT, glucose transporter type; GSK-3β, glycogen synthase kinase-3β; TNF, tumor necrosis factor; AGEs, advanced glycation end products; SOD, superoxide dismutase; GSH-Px, glutathione peroxidase; SIRP, signal regulatory protein; Nrf2, nuclear factor-erythrocyte-associated factor 2; mTOR, the target of rapamycin; S6k1, ribosomal protein S6 kinase 1; JNK, jun NH2-terminal kinase; w/v, weight/volume; w/w, weight/weight.

**Table 3:** The effects of tea on diabetes mellitus and its complications based on clinical trials.

Tea Types	Diseases Types	Study Types	Participants	Dose and Duration	Results	Ref.
Green tea	Diabetes mellitus	RCT	Patients with T2DM (N = 63)	0, 2, 4 cups per day	↓ Body weight, body mass index, waist circumference and systolic blood pressure.	[155]
Green tea	Diabetes mellitus and diabetic nephropathy	RCT	Patients with diabetes mellitus (N = 60)	2 capsules containing 1120 mg polyphenols per day for 20 weeks.	No significant effect on diabetes mellitus and diabetic nephropathy.	[158]
Green tea	T2DM and diabetic cardiomyopathy	RCT	Subjects with T2DM and lipid abnormalities (N = 92)	500 mg per day	↓ Triglyceride ↑ High density lipoprotein cholesterol ↑ Glucagon-like peptide 1 in the therapeutic arm	[156]
Green tea	Diabetic osteoporosis	RCT	Patients with diabetes mellitus (N = 35)	1120 mg polyphenols per day	↑ Bone mineral content ↓ PTH	[153]
Green tea	Bone turnover induced by diabetes mellitus	RCT	Patients with T2DM (N = 72)	500 mg per day	↓ Fasting serum osteocalcin ↓ FBG ↓ HbA1C	[154]
Black tea	T2DM and diabetic cardiovascular	N/A	Patients with T2DM (N = 46)	150, 300, 450, and 600 mL black tea during the weeks 1, 2, 3 and 4.	↓ Serum malondialdehyde ↓ Serum C-reactive protein ↑ Glutathione	[58]
Oolong tea	T2DM	N/A	Patients with T2DM	1500 mL per day	↓ Concentrations of plasma glucose and fructosamine	[73]
Green and black tea	T2DM	RCT	White persons (N = 49)	0, 375, or 750 mg per day for 3 months	No significant effect on T2DM.	[159]

N/A, not available; RCT, randomized, controlled clinical trial; FBG, Fasting blood glucose; T2DM, type 2 diabetes mellitus; PTH, parathyroid hormone; HbA1C, hemoglobin A1c.
